# Hexavalent Chromium Is Carcinogenic to F344/N Rats and B6C3F1 Mice after Chronic Oral Exposure

**DOI:** 10.1289/ehp.0800208

**Published:** 2008-12-31

**Authors:** Matthew D. Stout, Ronald A. Herbert, Grace E. Kissling, Bradley J. Collins, Gregory S. Travlos, Kristine L. Witt, Ronald L. Melnick, Kamal M. Abdo, David E. Malarkey, Michelle J. Hooth

**Affiliations:** National Toxicology Program, National Institute of Environmental Health Sciences, National Institutes of Health, Department of Health and Human Services, Research Triangle Park, North Carolina, USA

**Keywords:** anemia, cancer, hexavalent chromium, histiocytic cellular infiltration, National Toxicology Program, oral cavity, small intestine

## Abstract

**Background:**

Hexavalent chromium [Cr(VI)] is a human carcinogen after inhalation exposure. Humans also ingest Cr(VI) from contaminated drinking water and soil; however, limited data exist on the oral toxicity and carcinogenicity of Cr(VI).

**Objective:**

We characterized the chronic oral toxicity and carcinogenicity of Cr(VI) in rodents.

**Methods:**

The National Toxicology Program (NTP) conducted 2-year drinking water studies of Cr(VI) (as sodium dichromate dihydrate) in male and female F344/N rats and B6C3F1 mice.

**Results:**

Cr(VI) exposure resulted in increased incidences of rare neoplasms of the squamous epithelium that lines the oral cavity (oral mucosa and tongue) in male and female rats, and of the epithelium lining the small intestine in male and female mice. Cr(VI) exposure did not affect survival but resulted in reduced mean body weights and water consumption, due at least in part to poor palatability of the dosed water. Cr(VI) exposure resulted in transient microcytic hypochromic anemia in rats and microcytosis in mice. Nonneoplastic lesions included diffuse epithelial hyperplasia in the duodenum and jejunum of mice and histiocytic cell infiltration in the duodenum, liver, and mesenteric and pancreatic lymph nodes of rats and mice.

**Conclusions:**

Cr(VI) was carcinogenic after administration in drinking water to male and female rats and mice.

Hexavalent chromium [Cr(VI)] compounds are classified as human carcinogens [Cohen et al. 1993; International Agency for Research on Cancer (IARC) 1990; National Toxicology Program (NTP) 1998], based on increased incidences of lung cancers in animals and humans after inhalation exposures. Inhalation exposures occur primarily in occupational settings. The highest exposure to Cr(VI) occurs occupationally among workers involved in chrome plating, chromate production, and stainless steel welding.

As a result of industrial contamination, concentrations of Cr(VI) in the drinking water and soil may be higher than concentrations resulting from natural sources alone, causing ingestion of higher concentrations by populations residing near these locations than by the general population. A Cr(VI) concentration of 580 μg/L was found in a ground-water monitoring well in Hinkley, California (Pellerin and Booker 2000). Detectable levels of Cr(VI) have been reported in approximately 30% of the drinking water sources monitored in California, which has a 1 μg/L detection limit for purposes of reporting [California Department of Public Health (CDPH) 2007a]; 86% of those sources had peak concentrations of ≤ 10 μg/L. The U.S. Environmental Protection Agency (EPA) has set a maximum contaminant level of 100 μg/L for total chromium in drinking water (U.S. EPA 2003). The limit in California and numerous other states is 50 μg/L of total chromium in drinking water (CDPH 2007b).

The toxicity of Cr(VI) in humans and animals has been reviewed extensively (Agency for Toxic Substances and Disease Registry 2000; Costa 1997; Costa and Klein 2006; U.S. EPA 1998). We identified only one lifetime animal carcinogenicity study in the literature in which Cr(VI) was administered in the drinking water (Borneff et al. 1968). Further analysis of the data (Sedman et al. 2006) revealed that in three generations of female NMRI mice, the combined incidences of benign and malignant forestomach neoplasms were increased over controls; this study has several limitations, including high early mortality in the F_0_ generation as a result of ectromelia (mouse-pox) virus. A review of the few epidemiologic studies that evaluated populations that were exposed to Cr(VI) in drinking water or in soil or slag fill concluded that these studies did not provide definitive evidence of an increase in cancer incidence or mortality rates (Proctor et al. 2002). However, a retrospective mortality study in China found higher incidences of lung and stomach neoplasms in people living near a chromium smelting plant compared with the general population (Zhang and Li 1987); statistical analysis of these data supported this conclusion (Beaumont et al. 2008; Sedman et al. 2006).

Because of concerns over its presence in drinking water source supplies, its potential health effects, including carcinogenicity, and the lack of adequate carcinogenicity studies by the oral route, the California Congressional Delegation, California Environmental Protection Agency, and California Department of Health Services nominated Cr(VI) to the NTP for toxicity and carcinogenicity testing. We selected sodium dichromate dihydrate (SDD) for testing because it is the primary base material for the production of chromium compounds, is widely used in industrial applications, and is the most water-soluble chromate.

The NTP previously conducted 3-month toxicity studies of SDD administered in drinking water (NTP 2007). F344/N rats and B6C3F1 mice were exposed to 0, 62.5, 125, 250, 500, or 1,000 mg SDD/L. In these studies, both rats and mice had reduced body weight and water consumption and microcytic hypochromic anemia; the anemia was more severe in rats. Exposure-related histopathologic lesions included ulcers, epithelial hyper plasia, and squamous metaplasia in the glandular stomach of rats; epithelial hyperplasia of the duodenum of mice; and histiocytic cellular infiltration in the liver, duodenum, and pancreatic lymph node of rats and in the duodenum and mesenteric lymph node of mice.

We selected exposure concentrations for the 2-year studies of SDD after review of the 3-month toxicity study data. The highest exposure concentration for the 2-year studies in rats and mice was limited by toxicity observed in the 3-month studies (NTP 2007), and a wider spacing of exposure concentrations was selected to extend the dose–response curve. We added an additional low-exposure group [5 mg Cr(VI)/L] to the 2-year studies to provide a concentration closer to human exposure through contaminated drinking water. In this report we present the primary findings of the NTP chronic oral toxicity and carcinogenesis studies of Cr(VI) in male and female rats and mice; a more detailed report on these studies is available as an NTP technical report (NTP 2008a).

## Materials and Methods

### Chemical and dose formulations

SDD (CAS 7789-12-0) was obtained from Aldrich Chemical Company (Milwaukee, WI). The purity was determined using differential scanning calorimetry, titration of the dichromate ion with sodium thiosulfate and potassium ferrocyanide, speciation of the chromium ions using liquid chromatography/inductively coupled plasma-mass spectrometry, and potentiometric titrimetric analysis with sodium thiosulfate. Based on these analyses, the overall purity was ≥ 99.7%. The dose formulations were prepared approximately every 2 weeks by mixing SDD with tap water and stored at room temperature in sealed containers protected from light. The dose formulations were stable for 42 days under these conditions. Periodic analysis by ultraviolet spectroscopy with detection at 350–390 nm confirmed that all dose formulations varied by < 10% of the target concentrations.

### Animals and animal maintenance

The studies were conducted at Southern Research Institute (Birmingham, AL). Male and female F344/N rats and B6C3F1 mice were obtained from Taconic Farms (Germantown, NY). Rats and mice were quarantined for 14 days, and were 6–7 weeks of age at the beginning of the studies. Animals were distributed randomly into groups of approximately equal initial mean body weights and identified by tail tattoo. Rats and mice were housed one (male mice), three (male rats), or five (female rats and mice) to a cage. Feed (irradiated NTP-2000 wafers; Zeigler Brothers, Inc., Gardners, PA) and tap water were available *ad libitum*. Animals were killed by carbon dioxide asphyxiation.

Animal use was in accordance with the U.S. Public Health Service policy on humane care and use of laboratory animals and the *Guide for the Care and Use of Laboratory Animals* (National Research Council 1996). Animals were treated humanely and with regard for alleviation of suffering. These studies were conducted in compliance with the Food and Drug Administration Good Laboratory Practice Regulations (Food and Drug Administration 1987).

### Study design

Groups of 50 male and 50 female rats and mice were exposed to SDD in drinking water at concentrations of 0, 14.3, 57.3, 172, or 516 mg/L (male and female rats and female mice) or 0, 14.3, 28.6, 85.7, or 257.4 mg/L (male mice) for 105–106 weeks. Table 1 shows corresponding Cr(VI) concentrations. Water consumption was recorded weekly for the first 13 weeks and every 4 weeks thereafter, with each water consumption measurement covering a 7-day period. Animals were weighed initially, weekly for the first 13 weeks, at 4-week intervals thereafter, and at the end of the studies. Animals were observed twice daily and clinical findings were recorded at 4-week intervals beginning at week 5.

Complete necropsies and microscopic examinations were performed on all core study rats and mice. At necropsy, all organs and tissues were examined for grossly visible lesions, and all protocol-required tissues were fixed and preserved in 10% neutral buffered formalin (eyes were initially fixed in Davidson’s solution), trimmed and processed, embedded in paraffin, sectioned to a thickness of 4–6 μm, and stained with hematoxylin and eosin (H&E) for microscopic examination. For all paired organs (e.g., adrenal gland, kidney, ovary), samples from each organ were examined. The entire gastrointestinal tract was opened and potential lesions were collected for microscopic evaluation. Oral mucosa and tongue are not protocol-required tissues; however, because gross lesions in these tissues were diagnosed as neoplasms, the oral mucosa and tongue of all animals were evaluated histologically. Additional details regarding the pathology data generation, quality assurance review, and NTP pathology working group are available in the NTP technical report (NTP 2008a). Details of these review procedures have been described, in part, by Maronpot and Boorman (1982) and Boorman et al. (1985). For subsequent analyses of the pathology data, the decision of whether to evaluate the diagnosed lesions for each tissue type separately or combined was based on the guidelines of McConnell et al. (1986).

Hematology and clinical chemistry were evaluated in additional groups of 10–16 male rats and female mice on days 4 (male rats only) and 22 and at 3, 6, and 12 months. At these time points, rats and mice were anesthetized with CO_2_/O_2_, and blood was taken from the retroorbital sinus.

### Statistical methods

We estimated the probability of survival by the product-limit procedure of Kaplan and Meier (1958). Animals found dead of other than natural causes or missing were censored from the survival analyses; animals dying from natural causes were not censored. Statistical analyses for possible dose-related effects on survival used Cox’s (1972) method for testing two groups for equality and Tarone’s (1975) life table test to identify dose-related trends. All reported *p*-values for the survival analyses are two sided.

We used the poly-*k* test (Bailer and Portier 1988; Piegorsch and Bailer 1997; Portier and Bailer 1989) to assess neoplasm and nonneoplastic lesion incidence. This test is a survival-adjusted quantal-response procedure that modifies the Cochran-Armitage linear trend test to take survival differences into account; we used a value of *k* = 3 in the analysis of site-specific lesions. Tests of significance included pairwise comparisons of each exposed group with controls and a test for an overall exposure-related trend. We used continuity-corrected poly-3 tests in the analysis of lesion incidence, and reported *p*-values are one sided. Sub- and supralinear trends across doses for intestinal tumor rates were assessed with lack-of-fit tests for the linear regression of intestinal tumor rate on dose (Neter et al. 1996).

We analyzed hematology and clinical chemistry data, which typically have skewed distributions, using the modified (Dunn 1964; Williams 1986) nonparametric multiple comparison methods of Shirley (1977). We used Jonckheere’s test (Jonckheere 1954) to assess the significance of the dose-related trends and to determine whether a trend-sensitive test (Shirley 1977; Williams 1986) was more appropriate for pairwise comparisons than a test that does not assume a monotonic dose-related trend (Dunn 1964; Dunnett 1955). Before statistical analysis, we examined extreme values identified by the outlier test of Dixon and Massey (1957) and eliminated implausible values from the analysis.

## Results

### Oral cavity neoplasms in rats

Male and female rats showed significantly increased incidences of highly aggressive neoplasms of the squamous epithelium that lines the oral cavity (oral mucosa and tongue) at 516 mg/L (Table 2). Specifically, we observed increases for squamous cell carcinoma in the oral mucosa and for squamous cell papilloma or carcinoma (combined) of the oral mucosa or tongue of male and female rats at 516 mg/L. There were squamous cell carcinomas in the oral mucosa of two 172 mg/L female rats; this incidence exceeded the historical control ranges for drinking water studies and for all routes of administration (Table 2). We did not observe nonneoplastic lesions in the oral mucosa.

**Figure 1:**
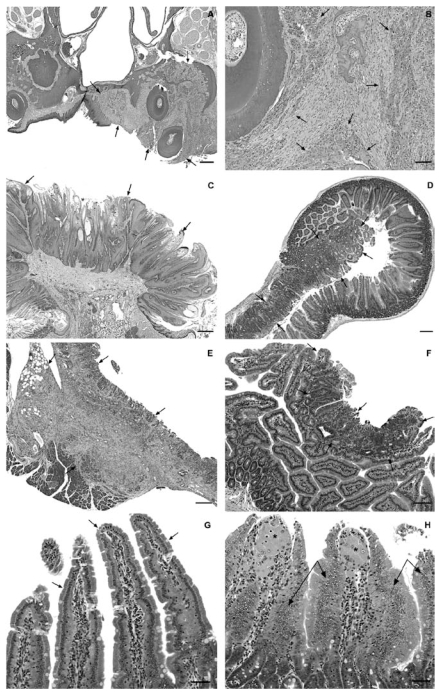
(*A* and *B*) Male rat given 516 mg/L SDD for 2 years. (*A*) Low magnification of a section of the nasal cavity demonstrating the location of squamous cell carcinoma (arrows) arising from the oral mucosa of the soft palate; the neoplasm has invaded the adjacent submucosal tissue and surrounded a molar tooth. (*B*) Higher magnification demonstrating the malignant features of the squamous cell carcinoma; islands and cords of dysplastic squamous epithelium (arrows) are surrounded by dense proliferative connective tissue stroma. (*C*) Female rat given 14.3 mg/L SDD for 2 years; squamous cell carcinoma projects from the dorsal mucosal surface of the tongue (arrows). (*D*) Female mouse given 516 mg/L SDD for 2 years; adenoma of the duodenum (arrows) has distorted and replaced a segment of the mucosa and protruded into the lumen. Normal mucosa is visible opposite the adenoma. (*E*) Male mouse given 257.4 mg/L SDD for 2 years; carcinoma of the duodenum (arrows) has effaced the mucosa, invading the submucosa, muscle layers, and pancreas. (*F*) Male mouse given 172 mg/L SDD for 2 years; focal hyperplasia of the duodenum (arrows) is present in the superficial mucosa. (*G*) Control female mouse; duodenum demonstrates normal microscopic anatomy. Note tall, slender villi (arrows) lined by a single layer of tall columnar epithelial cells. (*H*) Female mouse given 516 mg/L SDD for 2 years; diffuse hyperplasia is present in the duodenum. Duodenal villi are short, wide, and blunt and lined by hyperplastic epithelial cells that are piling up along the villi (arrows). Note histiocyte infiltrates expanding the lamina propria at the tips of the villi (asterisks). All sections are stained with hematoxylin and eosin. Bars: A and C, 500 μm; B and F, 100 μm; D and E, 200 μm; G and H, 50 μm.

Microscopically, the squamous cell carcinomas were highly invasive, irregular masses. Typically, the carcinomas appeared to originate in the oral mucosa of the palate adjacent to the upper molar teeth (Figure 1A, B); in some animals they invaded the tongue, Harderian gland, and the soft tissues surrounding the nose, and in one case it penetrated the maxilla and invaded the brain. The squamous cell papillomas of the oral mucosa and tongue were exophytic masses that projected from the mucosa and consisted of irregular papillary proliferations of mature squamous epithelium supported by a core of fibrovascular stroma (Figure 1C).

### Small intestine neoplasms and hyperplasia in mice

Both males and females showed a clear exposure concentration response for increased incidences of adenoma or carcinoma (combined) at all sites (combined) of the small intestine (duodenum, jejunum, or ileum; Table 3). These increases were significant at 85.7 and 257.4 mg/L in males and at 172 and 516 mg/L females, the two highest exposure concentrations in each sex. In addition, the incidence in 57.3 mg/L females exceeded the historical control ranges for drinking water studies and for all routes of administration (Table 3). These increases were driven primarily by significant increases in the incidences of adenoma of the duodenum in 257.4 mg/L males and in 172 and 516 mg/L females; the number of mice with multiple adenomas was also significantly increased at the high dose in both sexes (data not shown). In females, the incidence of carcinoma in the duodenum was significantly increased at 516 mg/L. In the jejunum, the incidence of adenoma was increased in 516 mg/L females. For both males and females, we tested the combined incidence of adenoma and carcinoma for departures from a linear dose response (data not shown). For males, the trend was linear, with no significant departure from linearity. For females, the response was supralinear with a significant departure from linearity.

Adenomas were discrete, broad based, focally extensive, plaquelike areas of proliferating glandular epithelium that thickened the mucosa and protruded into the lumen (Figure 1D). Carcinomas were sessile, plaque-like neoplasms distinguished from adenomas by extensive invasion and effacement of the mucosa, underlying submucosa, and muscle layers (Figure 1E).

Low incidences of focal epithelial hyperplasia occurred in the duodenum of exposed male and female mice (Table 4). Although the increased incidences were not exposure concentration related or statistically significant, we considered this lesion a preneoplastic lesion related to exposure to SDD because of its morphologic similarities to adenoma. Focal epithelial hyperplasias were focal areas of proliferating glandular epithelium distinguished from adenomas because they were smaller, less discrete, and confined to the superficial mucosal epithelium (Figure 1F).

The incidences of diffuse epithelial hyperplasia were significantly increased in the duodenum of all exposed groups of male and female mice (Table 4). In the jejunum, the incidence of diffuse epithelial hyperplasia was significantly increased in 516 mg/L females. In contrast to those of the controls (Figure 1G), the duodenums of exposed mice had short, broad, blunt villi and generalized mucosal hypercellularity that was particularly prominent in the villi (Figure 1H).

### Histiocytic infiltration in rats and mice

We found significant increases in histiocytic cell infiltration in the duodenum and mesenteric lymph node of rats and mice of both sexes, in the jejunum of female mice, in the liver of male and female rats and female mice, and in the pancreatic lymph node of female rats and male and female mice; these data are presented in detail in the NTP technical report (NTP 2008a).

The infiltrating histiocytes were morphologically similar in tissues of rats and mice. Histiocytic infiltrates were characterized by the presence of individual, small clusters, and sometimes syncytia of large histiocytes (macrophages) within the sinusoids of the liver and lymph nodes and the lamina propria at the tips of the duodenal and jejunal villi. In the lymph nodes, the histiocytic infiltrates occurred as expansive sheets that in some cases replaced much of the lymph node parenchyma. The biological significance and cause of the histiocytic cellular infiltrates are unknown.

### Hematology in rats and mice

In rats, an exposure-related decrease in mean cell volumes, mean cell hemoglobin concentrations, hematocrits, hemoglobin concentrations, and erythrocyte counts and increase in reticulocyte counts were indicative of erythrocyte microcytic, hypochromic, responsive anemia; these data are presented in detail in the NTP technical report (NTP 2008a). The anemia was most prominent early in the study (22 days to 3 months). Microscopic evaluations of blood smears demonstrated increased poikilocytes, erythrocyte fragments/schizocytes, keratocytes, erythrocyte hypochromia, and microcytes that suggested increased erythrocyte injury or turnover. This effect was more prominent on day 22 and at 3 months than at 6 or 12 months, which may have been either a result of the animals adapting to exposure or a result of the decreased Cr(IV) ingestion per unit body weight with longer exposure durations. Mice demonstrated a similar, but less severe, effect on erythron.

### In life effects in rats and mice

Survival, body weight, and water consumption data are presented in detail in the NTP technical report (NTP 2008a). Survival of exposed groups of male and female rats and mice was similar to that of the respective control groups. Mean body weights compared with controls were decreased in male and female rats and mice. In rats, mean body weights of 516 mg/L males and females were less than those of controls throughout the study and by the end of the study were 12% (males) or 11% (females) less than the controls. Mean body weights of 257.4 mg/L male mice were less than controls for the first 4 months of the study but were only slightly less (6%) by the end of the study. Mean body weights were less than the controls from months 3 to 12 in 172 mg/L female mice and from month 2 until the end of the study in 516 mg/L females. By the end of the study, mean body weights were < 8% of controls in 172 mg/L females and 15% less in 516 mg/L females.

We attributed the lower body weights partly to poor palatability of the dosed water and consequent reductions in water consumption rather than direct toxic effects of SDD exposure. Water consumption by male and female rats and mice exposed to the two highest concentrations was that by the controls throughout the study. No clinical findings were attributed to SDD exposure in rats or mice. When we adjusted water consumption for body weight (data not shown), dosed male and female rats and female mice drank approximately the same quantities of water per gram of body weight as did the controls after the first 20 weeks on study. Male mice exposed to 257.4 mg/L drank less water per gram of body weight than did the controls throughout the study.

## Discussion

Humans are exposed to Cr(VI) through ingestion of contaminated water and soil; however, few data exist on the oral toxicity and carcinogenicity of Cr(VI). The NTP conducted 3-month (NTP 2007) and 2-year (NTP 2008a) studies of SDD administered in the drinking water to F344/N rats and B6C3F1 mice, to provide data on the potential for toxic and carcinogenic effects after ingestion of Cr(VI).

Chronic administration of SDD in drinking water did not affect survival or produce clinical signs of toxicity in rats or mice. We observed exposure-related reductions in body weight gain and water consumption for rats and mice in the highest exposure groups and attributed these changes partly to poor palatability of the dosed water. Several lines of evidence suggest that the animals were not dehydrated, including analysis of the water consumption data normalized to body weight and the complete lack of clinical observations or hematologic or clinical chemistry effects (NTP 2008a) that typically indicate dehydration.

The NTP concluded that the exposure concentration-related significant increases in epithelial neoplasms of the upper alimentary tract (oral cavity) in male and female rats and of the lower alimentary tract (small intestine) in male and female mice provided clear evidence of carcinogenic activity of SDD in male and female rats and mice. We based this conclusion on the increased neoplasm incidences relative to concurrent controls and the rarity of these neoplasms (Tables 2 and 3) in historical controls. In both rats and mice, this conclusion was strengthened by similarities between the sexes. We observed no increases in nonneoplastic histopathologic lesions in either species suggestive of overt tissue damage due to the oxidant properties of Cr(VI).

We observed obvious species differences in the target tissues for the development of neoplasms between rats and mice. Of the 21 chemicals that have caused neoplasms of the oral cavity in NTP studies, none produced these neoplasms in male mice and only one, 1,2,3-trichloropropane (NTP 1993), produced oral cavity neoplasms in female mice, demonstrating a greater sensitivity to the development of oral cavity neoplasms in rats relative to mice. Although slightly more common in rats, exposure-related increases of small intestine neoplasms in NTP studies are relatively rare in both species. The 2-year study of captan (National Cancer Institute 1977) is the only other study performed by the NTP in B6C3F1 mice in which both benign and malignant intestinal neoplasms of epithelial origin have been definitely attributed to chemical exposure (Shackelford and Elwell 1999).

Although the induction of neoplasms after exposure to SDD was limited to the alimentary tract, other data, including the toxicity to the erythron, provided evidence of systemic exposure and toxicity in male and female rats and mice exposed to Cr(VI) for 2 years. We also observed these lesions in the 3-month studies (NTP 2007).

As part of the NTP 2-year studies on SDD (NTP 2008a) and chromium picolinate monohydrate (CPM) (NTP 2008b), which contains trivalent chromium [Cr(III)], total chromium content was determined in selected tissues and excreta of additional groups of male rats and female mice; these data will be presented in detail in an additional report. The goal of these studies was to examine the tissue uptake and distribution of Cr(VI) and Cr(III). Because Cr(VI) is reduced to Cr(III) both intracellularly and extracellularly and because analytical methods for the separate analysis of Cr(VI) or Cr(III) in biological samples are not available, the speciation of the tissue chromium after exposure to Cr(VI) was inferred by comparing total chromium concentrations in tissues of rats and mice exposed to similar doses of Cr(VI) or Cr(III). After oral exposure to Cr(VI), chromium accumulation was correlated with exposure concentration and duration in several tissues (NTP 2008a). Similar doses of Cr(VI) and Cr(III) resulted in significantly higher tissue chromium concentrations with Cr(VI), indicating that chromium was absorbed and distributed to tissues of rats and mice as Cr(VI); these data are consistent with previous studies (Costa 1997; Costa and Klein 2006). The tissue concentration data were consistent with linear or supralinear (decreasing rate of response with increasing dose) dose responses. In the present studies, neither the oral cavity nor the small intestine was collected for total chromium analysis. However, other reports suggest that Cr(VI) is also likely to be absorbed in the small intestine to a greater extent than Cr(III) (Donaldson and Barreras 1966; Febel et al. 2001).

Reduction of Cr(VI) to the less permeable and bioavailable Cr(III) is thought to occur primarily in the stomach, as a mechanism of detoxification. Gastric reduction has been hypothesized to be efficient, such that oral exposure to Cr(VI) would not result in toxicity or carcinogenicity, except perhaps in the stomach (De Flora 2000; De Flora et al. 1997; Proctor et al. 2002). Notably, in the 2-year study, no neoplasms or nonneoplastic lesions were observed in the forestomach or glandular stomach of rats or mice. However, the observed increases in neoplasms of the small intestine of mice and the toxicity to the erythron, histiocytic infiltration, and uptake of Cr(IV) into tissues of rats and mice suggest that, under the conditions of this study, at least a portion of the administered Cr(VI) was not reduced in the stomach. The significant disparity in the oral toxicity and carcinogenicity of Cr(VI) and Cr(III) in rodents, including the absence of increases in neoplasms or nonneoplastic lesions of the small intestine in rats or mice exposed to CPM (NTP 2008b), provides additional evidence that Cr(VI) is not completely reduced in the stomach and is responsible for the observed effects.

Recently, De Flora et al. (2008) have suggested that increases in neoplasms of the small intestine observed in mice in the present study are the result of saturation of the gastric reduction capacity. If such a threshold mechanism were to occur, the dose that saturated the reducing capacity would likely represent an inflection point on a sublinear dose response curve, with doses above the inflection point demonstrating an increasing rate of response per unit dose, because unreduced chromium would be transported into tissues. However, when we tested tissue concentration and mouse small intestine neoplasm data for linearity, data that were statistically nonlinear were supralinear (decreasing rate of response per unit dose).

A reduction capacity of about 84–88 mg Cr(VI)/day has been estimated for human gastric juice (De Flora et al. 1997). This estimate was based on reported values of human secretion of gastric fluid per day during fasting and after consuming three meals per day in combination with experimental data on reduction of Cr(VI)/mL of gastric juice produced during these periods. Similar data are not available for Cr(VI) reduction by mouse gastric juice. However, assuming that Cr(VI) reduction is equally effective in mice and humans and that gastric secretion scales across species by body weight^3/4^, then the Cr(VI) reduction capacity of gastric juice from a 50-g mouse would be approximately 0.4 mg/day (~ 8 mg/kg/day). This value is greater than all of the male mouse doses and is nearly equivalent to the average daily dose of Cr(VI) in the high-dose group of female mice in the 2-year drinking water study of SDD (Table 1). Collectively, the dose–response analysis and gastric reduction capacity calculations indicate that SDD induced neoplasms in the small intestine of mice at dose levels that did not exceed the estimated Cr(VI) reducing capacity for gastric juices in mice.

Cr(VI) is genotoxic in a number of *in vitro* and *in vivo* test systems (De Flora et al. 1990; IARC 1990); however, the mechanisms of genotoxicity and carcinogenicity are not fully understood. Because Cr(VI) as chromate structurally resembles sulfate and phosphate, it can be taken up by all cells and organs throughout the body through non-specific anion transporters (Costa 1997). Once inside the cell, indirect DNA damage may occur through the generation of oxygen radicals during intracellular reduction of Cr(VI) through the more reactive pentavalent and tetravalent chromium to Cr(III) (O’Brien et al. 2003); however, evidence of the role of reactive oxygen species in the genotoxicity of Cr(VI) is inconsistent (Chorvatovicova et al. 1991; O’Brien et al. 2003; Standeven and Wetterhahn 1991; Zhitkovich 2005). Cr(III), the final product of intracellular reduction of Cr(VI), has been shown to interact directly with DNA and other macromolecules to induce chromosomal alterations and mutational changes (O’Brien et al. 2003; Quievryn et al. 2006; Reynolds et al. 2007; Zhitkovich 2005). DNA adducts, DNA–protein cross-links, and DNA interstrand cross-links have all been identified as products of Cr(III)–DNA interactions. The relative contributions of the multiple, complex pathways of chromium-induced genotoxicity continue to be investigated.

In conclusion, the NTP 2-year study of SDD is the first and only lifetime study that clearly demonstrates the carcinogenicity of Cr(VI) in rats and mice after oral exposure. In addition, the hematology, histologic and tissue distribution data provide evidence of systemic exposure in rats and mice.

## Figures and Tables

**Table 1:** Concentrations in drinking water and average daily ingested doses of SDD and Cr(VI) after exposure for 2 years.

	Concentration in drinking water (mg/L)		Average daily ingested dose (mg/kg)	
Animals	SDD	Cr(VI)^a^	SDD^b^	Cr(VI)^c^
Male rats	0	0	0	0
	14.3	5	0.6	0.2
	57.3	20	2.2	0.8
	172	60	6	2.1
	516	180	17	5.9

Female rats	0	0	0	0
	14.3	5	0.7	0.2
	57.3	20	2.7	0.9
	172	60	7	2.4
	516	180	20	7.0

Male mice	0	0	0	0
	14.3	5	1.1	0.4
	28.6	10	2.6	0.9
	85.7	30	7	2.4
	257.4	90	17	5.9

Female mice	0	0	0	0
	14.3	5	1.1	0.4
	57.3	20	3.9	1.4
	172	60	9	3.1
	516	180	25	8.7

a Calculated using the drinking water concentration of SDD and the percent mass of Cr(VI) in SDD.

b Calculated using body weight and water consumption data.

c Calculated using the average daily dose of SDD and the percent mass of Cr(VI) in SDD.

**Table 2:** Squamous cell neoplasms of the oral cavity (oral mucosa and tongue) of F344/N rats exposed to SDD for 2 years in drinking water.

	Historical control incidence^a^ (range)		Incidence/no. of animals necropsied (survival-adjusted percent incidence)^b^				
Tissue/neoplasm	Drinking water	All routes	0 mg/L	14.3 mg/L	57.3 mg/L	172 mg/L	516 mg/L
Males							

Oral mucosa							
Carcinoma	0/350	5/1,499 (0–2%)	0/50#	0/50	0/49	0/50	6/49 (13.6)*
Tongue							
Papilloma	ND^c^	ND	0/50	0/50	0/49	0/50	1/49 (2.3)
Carcinoma	ND	ND	0/50	1/50 (2.4)	0/49	0/50	0/49
Oral mucosa or tongue							
Papilloma or carcinoma (combined)	1/350 (0–2%)	10/1,499 (0–2%)	0/50#	1/50 (2.4)	0/49	0/50	7/49 (15.7)**

Females							

Oral mucosa							
Carcinoma	0/300	5/1,400 (0–2%)	0/50#	0/50	0/50	2/50^d^ (4.6)	11/50 (23.9)#
Tongue							
Papilloma	ND	ND	1/50 (2.2)	1/50 (2.3)	0/50	0/50	0/50
Carcinoma	ND	ND	0/50	0/50	0/50	1/50 (2.3)	0/50
Oral mucosa or tongue							
Papilloma or carcinoma (combined)	4/300 (0–2%)	15/1,400 (0–6%)	1/50 (2.2)#	1/50 (2.3)	0/50	2/50^e^ (4.6)	11/50 (23.9)**

ND, not determined.

a The NTP historical database contains all studies that use the NTP-2000 diet with histopathology findings completed within the most recent 5-year period, including the present study.

b Calculated using the poly-3 test.

c Historical control incidences are not determined for the tongue because it is not an NTP protocol-required tissue.

d The incidence exceeded the historical control range for both drinking water studies and all routes but was not significantly increased compared with the concurrent control.

e The incidence exceeded the historical control range for drinking water studies but was not significantly increased compared with the concurrent control.

* p ≤ 0.05,

** p ≤ 0.01, and

# p ≤ 0.001 compared with the control group by poly-3 test or a significant trend if assigned to a control group.

**Table 3:** Epithelial neoplasms of the small intestine in B6C3F1 mice exposed to SDD in drinking water for 2 years.

	Historical control incidence^a^ (range)						
Tissue/neoplasm	Drinking water	All routes	Incidence/no. of animals necropsied (survival-adjusted % incidence)^b^				
Males							

Concentration (mg/L)			0	14.3	28.6	85.7	257.4
Duodenum							
Adenoma (includes multiple)	6/299 (0–6%)	9/1,549 (0–6%)	1/50 (2.2)#	0/50	1/50 (2.3)	5/50^c^ (10.8)	15/50 (32.9)#
Carcinoma	1/299 (0–2%)	3/1,549 (0–4%)	0/50*	0/50	0/50	2/50^d^ (4.3)	3/50^c^ (6.8)
Jejunum							
Adenoma	0/299	1/1,549 (0–2%)	0/50**	0/50	0/50	0/50	3/50^c^ (6.8)
Carcinoma (includes multiple)	5/299 (0–4%)	25/1,549 (0–8%)	0/50	2/50 (4.5)	0/50	1/50 (2.2)	2/50 (4.6)
Duodenum, jejunum, or ileum (combined)							
Adenoma	6/299 (0–6%)	10/1,549 (0–6%)	1/50 (2.2)#	1/50 (2.3)	1/50 (2.3)	5/50^c^ (10.8)	17/50 (37.2)#
Carcinoma	6/299 (0–4%)	30/1,549 (0–8%)	0/50*	2/50 (4.5)	1/50 (2.3)	3/50^d^ (6.5)	5/50 (11.4)*
Adenoma or carcinoma (combined)	11/299 (0–10%)	39/1,549 (0–10%)	1/50 (2.2)#	3/50 (6.8)	2/50 (4.6)	7/50 (15.1)*	20/50 (43.8)#

Females							

Concentration (mg/L)			0	14.3	57.3	172	516
Duodenum							
Adenoma (includes multiple)	1/350 (0–2%)	3/1,648 (0–2%)	0/50#	0/50	2/50^c^ (4.2)	13/50 (27.8)#	12/50 (25.2)#
Carcinoma	0/350	1/1,648 (0–2%)	0/50#	0/50	0/50	1/50^d^ (2.1)	6/50 (12.6)*
Jejunum							
Adenoma (includes multiple)	0/350	0/1,648	0/50**	1/50^c^ (2.2)	0/50	2/50^c^ (4.3)	5/50 (10.6)*
Carcinoma	2/350 (0–2%)	5/1,648 (0–2%)	1/50 (2.2)	0/50	2/50^c^ (4.2)	2/50^c^ (4.3)	1/50 (2.1)
Duodenum, jejunum, or ileum (combined)							
Adenoma	1/350 (0–2%)	3/1,648 (0–2%)	0/50#	1/50 (2.2)	2/50^c^ (4.2)	15/50 (32.0)#	16/50 (33.7)#
Carcinoma	3/350 (0–2%)	8/1,648 (0–2%)	1/50 (2.2)#	0/50	2/50^c^ (4.2)	3/50^c^ (6.4)	7/50 (14.7)*
Adenoma or carcinoma (combined)	4/350 (0–4%)	11/1,648 (0–4%)	1/50 (2.2)#	1/50 (2.2)	4/50^c^ (8.3)	17/50 (36.3)#	22/50 (45.9)#

a The NTP historical database contains all studies that use the NTP-2000 diet with histopathology findings completed within the most recent 5-year period, including the present study.

b Calculated using the poly-3 test.

c The incidence exceeded the historical control range for both drinking water studies and all routes but was not significantly increased compared with the concurrent control.

d The incidence exceeded the historical control range for drinking water studies but was not significantly increased compared with the concurrent control.

* p≤ 0.05,

** p ≤ 0.01, and

# p ≤ 0.001 compared with control group by poly-3 test or a significant trend if assigned to a control group.

**Table 4:** Focal and diffuse hyperplasia of the small intestine in B6C3F1 mice exposed to SDD in drinking water for 2 years.

Tissue	Incidence/no. of animals necropsied (mean severity)^a^				
Males					

Concentration (mg/L)	0	14.3	28.6	85.7	257.4
Duodenum					
Epithelium, hyperplasia					
Focal	0/50	0/50	0/50	1/50 (3.0)	2/50 (3.5)
Diffuse	0/50	11/50** (2.0)	18/50** (1.6)	42/50** (2.1)	32/50** (2.1)

Females					

Concentration (mg/L)	0	14.3	57.3	172	516
Duodenum					
Epithelium, hyperplasia					
Focal	0/50	0/50	1/50 (2.0)	2/50 (3.0)	0/50
Diffuse	0/50	16/50** (1.6)	35/50** (1.7)	31/50** (1.6)	42/50** (2.2)
Jejunum					
Epithelium, hyperplasia					
Diffuse	0/50	2/50 (2.0)	1/50 (2.0)	0/50	8/50** (1.9)

a Mean severity: 1, minimal; 2, mild; 3, moderate; 4, marked.

** p ≤ 0.01 by poly-3 test.
